# Lower polyunsaturated fatty acid levels and FADS2 expression in adult compared to neonatal keratinocytes are associated with FADS2 promotor hypermethylation

**DOI:** 10.1016/j.bbrc.2022.02.055

**Published:** 2022-04-23

**Authors:** C. Pararasa, D.J. Messenger, K.E. Barrett, D. Hyliands, D. Talbot, M.I. Fowler, T. Kawatra, D.A. Gunn, F.L. Lim, L.J. Wainwright, G. Jenkins, H.R. Griffiths

**Affiliations:** a College of Health and Life Sciences, Aston University, Aston Triangle, Birmingham, B4 7ET, UK; b Unilever R&D, Colworth Science Park, Sharnbrook, Bedfordshire, MK44 1LQ, UK; c Faculty of Medicine, Health and Life Sciences, Swansea University, SA2 8PP, UK

**Keywords:** Fatty acid, Epigenetic, mRNA, GC-MS, Keratinocytes, SMARCA4, Fatty acids, FA, Fatty acid Desaturase, FADS, Elongase, ELOVL, Normal Human Epidermal Keratinocytes, NHEK

## Abstract

Keratinocytes produce lipids that are critical for the skin barrier, however, little is known about the impact of age on fatty acid (FA) biosynthesis in these cells.

We have examined the relationship between keratinocyte FA composition, lipid biosynthetic gene expression, gene promoter methylation and age.

Expression of elongase (ELOVL6 and 7) and desaturase (FADS1 and 2) genes was lower in adult versus neonatal keratinocytes, and was associated with lower concentrations of n-7, n-9 and n-10 polyunsaturated FA in adult cells. Consistent with these findings, transient FADS2 knockdown in neonatal keratinocytes mimicked the adult keratinocyte FA profile in neonatal cells.

Interrogation of methylation levels across the FADS2 locus (53 genomic sites) revealed differential methylation of 15 sites in neonatal versus adult keratinocytes, of which three hypermethylated sites in adult keratinocytes overlapped with a SMARCA4 protein binding site in the FADS2 promoter.

## Highlights

• Primary keratinocytes from adult donor skin synthesise less unsaturated fatty acids compared to keratinocytes from neonates.
• FADS2 knockdown in neonatal keratinocytes switches cellular fatty acid composition to mimic adult keratinocytes.
• In adult keratinocytes, the SMARCA4 binding site in the FADS2 promoter is hypermethylated.
• Epigenetic regulation at the SMARCA4 binding site may affect fatty acid composition in skin cells.

## 1 Introduction

The skin surface is covered with a thin lipid layer, synthesised and secreted by sebocytes and keratinocytes, which provides an epidermal barrier to protect against infection and injury.

The cellular-derived surface lipids that make up the skin epidermal barrier function consist of phospholipids, ceramides and cholesterol esters, released by keratinocyte lamellar bodies [1]. Fatty acids (FA) are important constituents of these complex lipids and contribute 15% of total lipid content within and on the skin surface [2,3]. Polyunsaturated FA, particularly linoleic acid secreted by keratinocytes into the surface hydrophobic film may be metabolised further by resident skin surface microbial flora; the products, antibacterial monohydroxy FA, target non-host pathogenic organisms and provide further antimicrobial protection to the host [4,5]. In this way, surface lipids regulate both barrier permeability and risk for infection [6].

The cellular FA lipidome is determined by both dietary FA supply and metabolic enzyme activity, regulated within a complex feedback loop [7]. Two major classes of FA biosynthetic enzymes are the elongases (encoded by the ELOVL genes) and the desaturases. Several discrete FA-metabolising enzyme deficits e.g. in elongation enzymes (ELOVL) and desaturases have been reported to impair skin barrier function [8]. Disruption of elongase and desaturase genes contributes to skin pathology and highlights the importance of FA biosynthesis for skin function [9].

There is a profound reduction in the total lipid content in skin from older adults [10]. This is believed to contribute to dryness, increased susceptibility to infection and impaired wound healing in response to injury. Although very long chain lipids are essential for creating an effective barrier [11], it is not known whether their biosynthetic machinery is affected by development or age.

Epigenetic regulation of FA biosynthesis has been described. Consistent with the hypothesis that a FA profile change with age is due to altered biosynthesis, a recent study has shown that hypermethylation of CpG islands in the promoter region of the elongase gene ELOVL2 is associated with age in a number of cell types, particularly those with high proliferation rates [12].

Here we have investigated the hypothesis that FA composition and concentration are different in keratinocytes from adult compared to neonatal donors due to specific differences in expression of FA biosynthesis gene expression. Our aim was to understand whether any molecular changes in FA profile in keratinocytes from different age donors are associated with epigenetic modification of specific FA biosynthesis gene promoter region.

## 2 Methods

### 2.1 Cell culture

Normal human epidermal keratinocytes (NHEK) from Lonza, Belgium were grown in serum-free keratinocyte KGM media supplemented with KGM Gold bullet kit (Lonza, Belgium) containing 60 μM calcium. Media was replaced every 48 h. To eliminate any passage-induced effects, all primary neonatal and adult NHEK were studied at passage 2. Senescence was detectable from passage 5.

### 2.2 Real-time PCR

Messenger RNA was extracted from NHEK using RNeasy mini plus kit (Qiagen) and RNA (500 ng) was converted to cDNA using a qScript cDNA synthesis kit (Quanta Bioscience). Quantitect primers were used for ELOVL1, 2, 3, 4, 5, 6, and 7, SCD-1, FADS1 and FADS2 expression, with YWHAZ as a housekeeping gene (Qiagen), using a Stratagene Mx3000P system. The relative gene expression was calculated against the input RNA.

### 2.3 FA analysis of normal human epidermal keratinocytes

Cell lysates (normalized to the lowest protein concentration) were prepared from proliferating NHEK cells (95% confluent) and spiked with 0.1 mg C13:0 and C17:0 before extraction of lipids as previously described by us [13]. After derivatisation with 0.2 ml toluene, 1.5 ml methanol, 0.3 ml 8% HCl at 100 °C for 1 h, the FA methyl esters were extracted into hexane (10 μl) prior to analysis by GC-MS. Reproducibility was monitored by C13:0 and C17:0 recovery; intra-batch variation was <5%.

### 2.4 Gas chromatography (GC) –mass spectrometry (MS) analysis of FAMEs

Methylated lipids (1 μl) were injected on an Agilent 6890 GC in 0.9 ml/min helium (20:1 split) coupled to an Agilent 5973 MS and FAs were separated on an OmegaWax 250 column (30 m × 0.25 mm x 0.25 μm; Supelco). Peaks were identified using standards from Supelco (37 FAME mix, Sigma-Aldrich), Larodan (Solna, Sweden) and by comparing mass spectral data to the NIST library. Peaks were quantitated as peak area and expressed relative to C16:0.

### 2.5 Gene knockdown

Accell Smartpool siRNA was purchased from Dharmacon (GE Lifescience, UK) for targeting of ELOVL6, FADS1 and FADS2 genes, with positive (GAPDH) and vehicle control siRNA. Neonatal NHEK (n = 5 independent donors) were grown until 60% confluence, then treated with either ELOVL6, FADS1 or FADS2 siRNA (1 μM) as per manufacturer's protocol and grown for a further 96 h at which point media was changed and siRNA introduced for a further 72 h. Gene knockdown was confirmed using RT-PCR.

### 2.6 Methylomics

DNA was extracted using a DNAeasy blood and tissue kit (Qiagen). The genomic DNA was quantitated using Quant-iT™ PicoGreen® dsDNA Assay Kit (Life-Technologies), following the manufacturer's protocol using the high-range standard curve. Bi-sulphite conversion of the DNA and detection of methylated and non-methylated DNA was performed by Nxt-DX (Belgium). Genomic DNA was bisulfite-treated using the EZ DNA methylation kit (Zymo Research, Irvine) according to the manufacturer's specifications. The samples were then processed with the Infinium Methylation EPIC BeadChip kit according to the manufacturer's instructions. Quality checks were performed using density bean plots, probe detection p-values, and PCA analyses – no samples failed these checks. Beta-values are defined as beta = methylated/(methylated + unmethylated + offset); the offset of 100 is chosen to avoid dividing with small values. The beta values were normalized using SWAN normalization, and the M-values then calculated as M = logit(beta) = log(methylated/unmethylated). The M-values were used for statistical analyses in Genespring (version 14.8) and beta values used for subsequent filtering. In a targeted methylomics strategy, DNA methylation levels at 53 genomic sites located in and around the FADS2 gene were interrogated.

### 2.7 Statistical analysis

Statistical analysis was performed using GraphPad Prism (GraphPad, San Diego, USA) Qlucore (Qlucore, Lund, Sweden) or GeneSpring (Agilent Genomics, Santa Clara, USA) where stated. For the methylation data, replicate sample data was averaged, imported into Genespring and a moderated T-test performed on the data for probes annotated to FADS2 gene (53 probes). A false discovery rate (FDR) of p < 0.05 was considered significant. Binding sites for transcription factor binding proteins were assessed in the UCSC browser (https://genome.ucsc.edu/cgi-bin/hgGateway) by blasting the source methylation probe sequences and visually inspecting for overlap of the resulting genomic location with OReg Anno transcription factor binding site (TFBS) data [14].

### 2.8 Data availability

The datasets generated during and/or analysed during the current study are available from the corresponding author on reasonable request.

## 3 Results

### 3.1 Donor age-associated differences in the FA lipidome

**Fig. 1:**
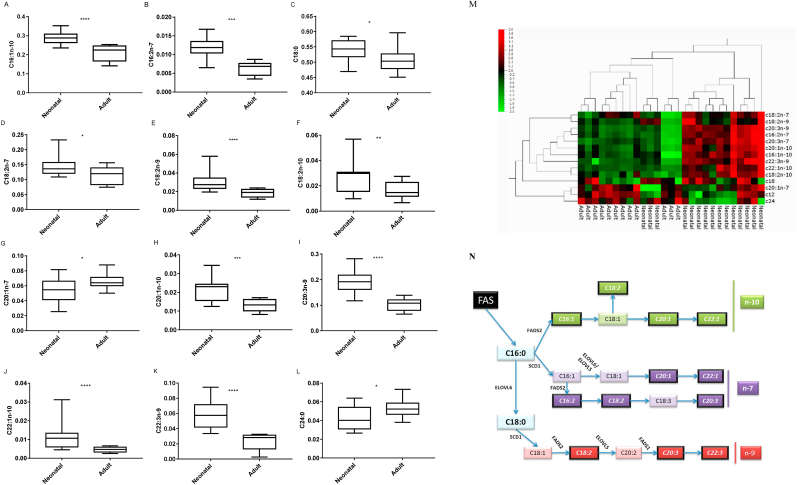
FA analysis of neonatal and adult NHEK. Data represent the analysis of extracts from 5 neonatal and 4 adult donor keratinocytes, grown in triplicate to confluence and extracted for independent analyses, p < 0.05 (A–L). Heat map visualisation of discrete FA profiles from neonatal and adult NHEK (M). Only FA where significant differences were seen according to donor age are included p < 0.05. Summary of the n-7, n-9, and n-10 FA synthetic pathways (N). The FAs in italics and outlined in black were found to be significantly reduced in adult cells compared to neonatal cells.

A heat map of lipid levels (Fig. 1M) confirms discrete FA profiles for neonatal and adult NHEK. Adult keratinocytes had greater C20:1n-7 and C24:0 levels (∼21% and 25% respectively, p < 0.05) compared to neonatal cells (Fig. 1G, L). Conversely, C16:1n-10, C16:2n-7, C18:0, C18:2n-7, C18:2n-9, C18:2n-10, C20:1n-10, C20:3n-9, C22:1n-10, and C22:3n-9 levels were lower in adult NHEK (∼26%, 44%, 5%, 19%, 37%, 40%, 38%, 45%, 60%, and 60% of neonatal NHEK, p < 0.0001–0.05; Fig. 1A–F, H-K). PCA analysis illustrates that age rather than gender is the principal driver of NHEK lipid profiles (Supplementary Fig. 1A). The FA biosynthetic pathway is illustrated in Fig. 1N, showing the lipids (heavy-lined boxes) that are different according to donor age and the enzymes regulating their synthesis.

### 3.2 FA synthetic pathway gene expression

**Fig. 2:**
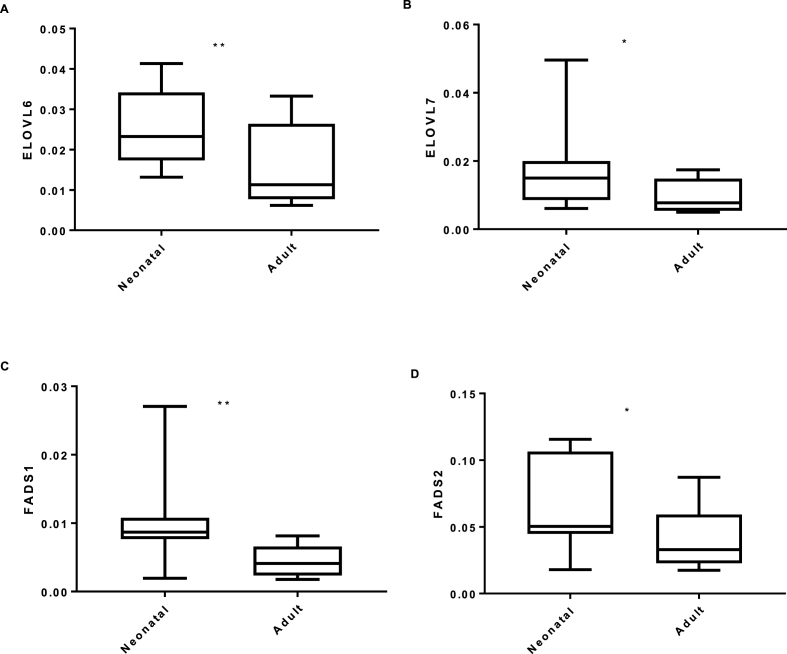
RT-PCR analysis of FA synthetic gene expression from proliferating neonatal and adult NHEK. Expression was calculated relative to housekeeper YWAHZ and is displayed as relative expression; ∗ = p < 0.05, ∗∗ = p < 0.01.

ELOVL6 and 7 were expressed at lower levels in adult NHEK compared to neonatal NHEK (p < 0.01 and p < 0.05; Fig. 2). Analysis of FA desaturase gene expression confirmed that FADS1 and FADS2 expression in adult NHEK was lower than in neonatal NHEK (p < 0.01 and p < 0.05; Fig. 2).

### 3.3 Knock-down of FA biosynthetic genes in NHEK

**Fig. 3:**
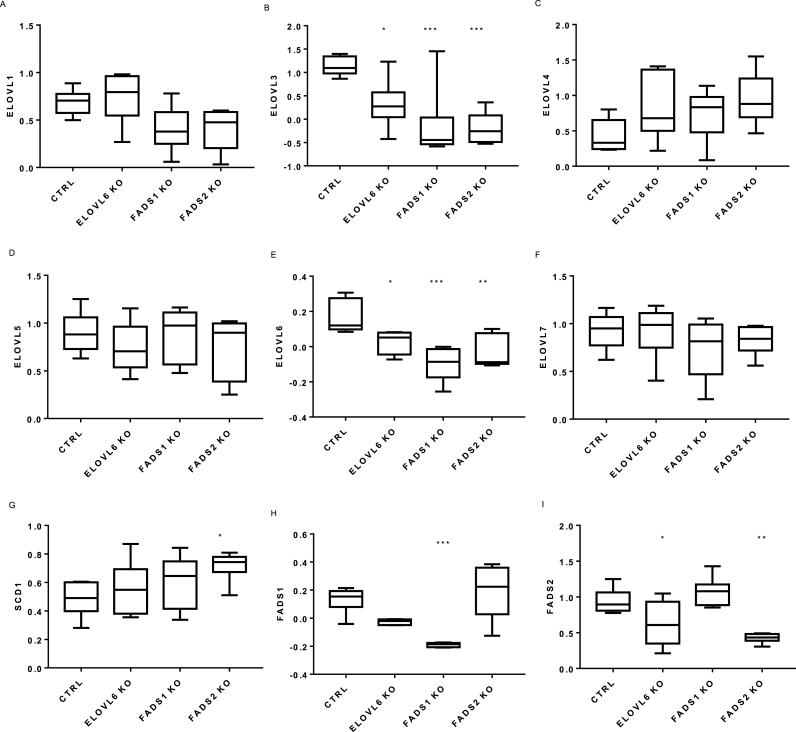
siRNA-mediated lipid biosynthetic gene knockdown in neonatal NHEK. Neonatal NHEK cells at 60% confluence were either untreated, treated with vehicle control or specific ELOVL6, FADS1 or FADS2 siRNA. Data is expressed as mean of 5 individual neonatal NHEK donor cells. RT-PCR analysis was relative to housekeeper YWAHZ ∗ = p < 0.05, ∗∗∗∗ = p < 0.0001 compared to untreated proliferating NHEK.

The FAs which are lower in adult compared to neonatal NHEK are produced by ELOVL6, FADS1 and FADS2 activity. These genes were targeted by gene silencing in neonatal NHEK to determine their potential contributions to NHEK lipid profiles. Knockdown of ELOVL6, FADS1 and FADS2 genes was performed and confirmed by qPCR, with ∼50% reduction in the level of expression of ELOVL6 and FADS2 and complete absence of FADS1 (Fig. 3E, H, I). Specificity of the siRNA knockdown was confirmed by measuring expression of β-actin and GAPDH, eliminating the possibility of a global reduction in gene expression (Supplementary Fig. 2). Despite specificity of the primers as confirmed by BLAST search, independent siRNA knockdown of ELOVL6, FADS1 and FADS2 also affected other lipid biosynthetic genes and caused a significant reduction in ELOVL3 expression (Fig. 3). Similarly, independent siRNA knockdown of FADS1 and FADS2 resulted in a significant off-target reduction in ELOVL6 expression. The reciprocal experiment confirmed that ELOVL6 knockdown also led to a reduction in FADS2 expression. Conversely, FADS1 knockdown was specific to cells treated with FADS1 siRNA. In addition to the expected knockdown of FADS2, an increase in SCD1 expression was observed when FADS2 expression was inhibited.

### 3.4 Keratinocyte FA profile after knock-down of ELOVL6, FADS1 and FADS2 expression in NHEK

**Fig. 4:**
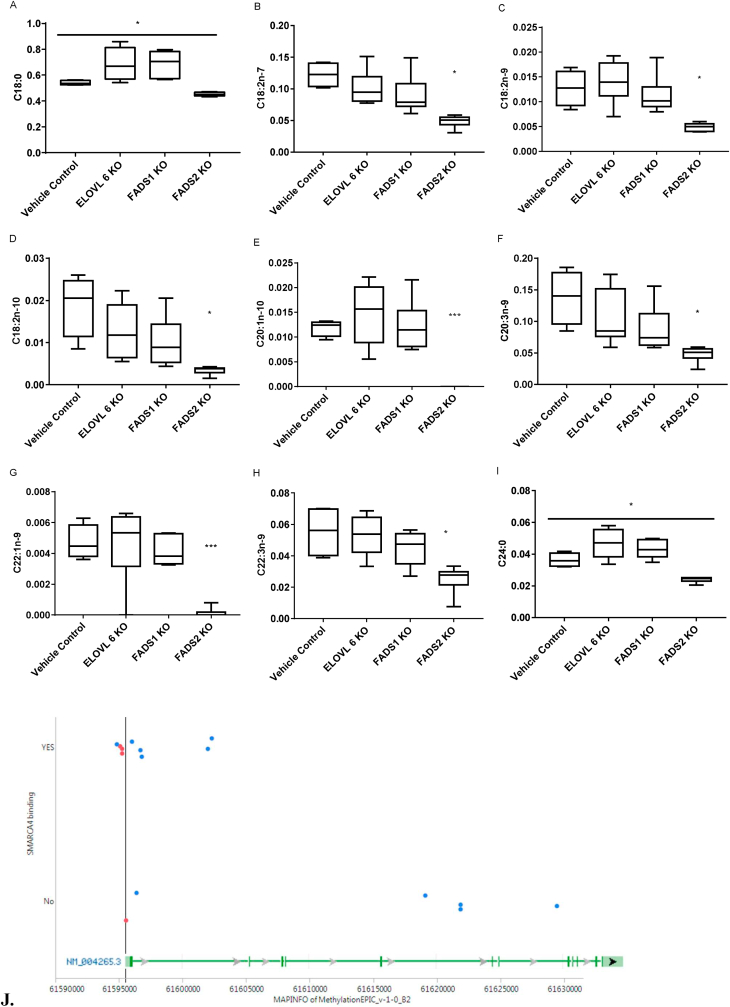
A-I. FA analysis of neonatal NHEK following transient knockdown of ELOVL6, FADS1 and FADS2. Data represent the analysis of extracts from 5 neonatal and 4 adult donor keratinocytes, which were each grown in triplicate, treated with siRNA or control ∗ = p < 0.05. J. Location of genomic sites differentially methylated in adult compared to neonatal keratinocyte cells. The genetic structure of the FADS2 isoform 1 gene is represented by the green bars and arrows at the bottom of the Figure (from NCBI, genome build 37). The sites overlapping with SMARCA4 binding sites are represented by points in the upper section of the Figure. Sites less than 200 bp of the FADS2 transcriptional start site (vertical line) are represented by red points. Map information (x-axis) was taken from the Illumina annotations and the visualisation generated in JMP (version 11.0.0). (For interpretation of the references to colour in this figure legend, the reader is referred to the Web version of this article.)

The contribution of ELOVL6, FADS1 and FADS2 expression for NHEK FA levels was analysed in NHEK after siRNA knockdown (Fig. 4). Only FADS2 knockdown led to a consistent decrease in FA synthesis in NHEK and eight of the ten FAs found at lower concentrations in adult NHEK were reduced in neonatal NHEK following FADS2 siRNA treatment. C20:1n7 levels were unaffected by any of the knockdowns tested, however, ELOVL6 and FADS1 knockdown in neonatal NHEK increased both C18:0 (not seen in adult NHEK) and C24:0 (found at increased levels in adult NHEK).

### 3.5 Targeted methylomics

A total of 15 significantly differentially methylated sites in FADS2 were identified, and all but one had a delta beta greater than 5% between adult and neonatal cells. Only one site was hypomethylated in the adult samples and was located approximately 23 Kbp downstream of the FADS2 isoform 1 (NM_004265) transcriptional start site (TSS). All other hypermethylated sites demonstrated a similar difference (i.e. delta beta) between the neonatal and adult female samples as they did between the neonatal and adult male sample. In addition, 4 of the sites were located in the FADS2 promoter (<200 bp upstream of the TSS) and 9 probes (all detecting hypermethylated sites in adult cells) overlapped with SMARCA4 binding sites (Table 1; Fig. 4J); 3 of these 9 sites were within the FADS2 promoter region. PCA analysis illustrates that age rather than gender is the principal driver of differential hypermethylation in the SMARCA4 promoter locus of the FADS2 gene (Supplementary Fig. 1B).

**Table 1:** TF Binding sites overlap significantly across differentially methylated probes for ELOVL5 & 6, FADS1 & 2.

Gene	ELOV5	ELOVL6	FADS1	FADS2
Total Probes	N:6	N:3	N:3	N:13
SMARCA4	2	3	1	8
HNF4A	1	.	.	.
CEBPA	1	.	.	.
SPI1	.	1	.	2
FOS	.	1	.	.
CTCF	.	1	.	3
STAT1	.	.	1	.
TFAP2C	.	.	1	.
ZNF263	.	.	.	2
RBL2	.	.	.	4
TAL1	.	.	.	1
ETS1	.	.	.	1
GATA2	.	.	.	1
FLI1	.	.	.	1
ELF1	.	.	.	1
Total probes overlapping with TFBS	3/6	3/3	1/3	9/13

## 4 Discussion

Our work has shown that NHEK from healthy adults have lower unsaturated FA levels and reduced expression of FADS1 and 2, ELOVL6 and 7 compared to neonatal NHEK. Only FADS2 knockdown in neonatal NHEK was able to recapitulate the adult NHEK FA profile, indicating that lower expression of FADS2 is a major contributor to the differences in FA composition. To understand why FADS2 expression was lower in adult NHEK, we explored epigenetic modification of promotor and identified that binding sites for the helicase, SMARCA4, were hypermethylated in the promoter of FADS2 in adult NHEK.

To our knowledge, this is the first report of differential FA level and gene expression with age in skin cells. An earlier study described FADS2 gene expression in young, old, ultraviolet (UV)-protected and UV-exposed skin with FADS2 being consistently down-regulated in intrinsic skin ageing and photo-ageing; however, functional effects on lipid composition were not explored [15].

Lower FADS2 expression in skin is described in inflammatory skin conditions (acne, dermatitis, psoriasis). Dermatitis also develops after FADS2-targeted disruption in the skin [10] further supporting the significance of FADS2 expression for normal skin function.

The epigenome becomes increasingly divergent with age due increasing exposure to stochastic events. In general, a progressive genome demethylation is observed with age [16], however, a limited gene-specific CpG dinucleotide hypermethylation sites have also been reported [17]. In mouse skin, the expression of DNA methyl transferase (Dnmt)3a, Dnmt3b and Tet2 declines significantly with age but genome-wide DNA methylation analysis identified both age-dependent gene promoter hyper- and hypo-methylation [18].

Previously, common age-associated hypermethylation sites have been described for ELOVL2, SFMBT1, KLF14, PENK and FHL2 despite differences in sample tissue types or age distributions [19,20]. However, ELOVL2 is not expressed in human NHEK, therefore its methylation status was not relevant here. Instead, we explored the FADS2 promoter and showed hypermethylation in adult compared to neonatal NHEK.

Detailed analysis of the FADS2 promoter confirmed that SMARCA4 binding sites overlapped with nine FADS2 probes, and three methylation sites overlapped with a SMARCA4 binding site. All nine FADS2 probes showed increased methylation levels in adults, which normally contributes to lower gene expression. This is in keeping with the lower FADS2 mRNA that we observed in adult NHEK.

SMARCA4 is a master regulator of epidermal differentiation, mediating the effects of p63 in skin [21]. As a helicase it promotes chromatin relaxation for gene transcription. Hypermethylation of the FADS2 promoter is predicted to reduce SMARCA4 binding, reducing DNA unwinding and lower transcription of the downstream FADS2 gene.

In skin, the ELOVLs are responsible for extending FA chain length to create a functionally diverse unsaturated FA pool upstream and downstream from FADS2. While we identified lower ELOVL7 in adult NHEK, which normally catalyses the extension of saturated FA chains, this was not paralleled by any differences between adult and neonatal NHEK FA levels. ELOVL6 is important for C18:1n7 production. Despite a lack of difference between neonatal and adult NHEK C18:1n7 levels, we investigated the effect of ELOVL6 knockdown. We found that its regulation was tightly coupled to FADS1 and 2, indicating that FADS gene expression differences were likely to be the major determinates of FA profiles.

The observed lower levels of PUFA in adult NHEK compared to neonatal NHEK are attributed to lower FADS expression (Fig. 1). In support, another skin biopsy study, including mixed cell populations, identified reducing expression of FADS1, ELOVL3, 4 and 5 with increasing age in 100 post-mortem tissues from adults aged 39–85 [22].

C16:1n-10 is a secretion product from sebaceous cells following C16:0 desaturation by FADS2 or from linoleic acid metabolism [23]. C16:1n-10 may undergo further elongation and desaturation forming C18:2n-10. In the present study, novel n-10 FA, C20:1n-10 and C22:1n-10, were putatively identified in NHEK by GC-MS. To rule out sebocytes as potential contaminants in NHEK, the expression of a sebocyte marker, keratin-7, was analysed in NHEK (Supplementary Table 1). The Ct values (in the order of 10^15^ times lower than other FA genes) for keratin-7 were negligible, and therefore we suggest that NHEK possess a novel n-10 FA profile.

ELOVL6 is also essential for C18:1n-9 synthesis and C18:1n-9 can be further metabolised by FADS2 in keratinocytes to form other n-9 FAs [24]. Coordinated induction of FA elongase and desaturase activity is required for balanced synthesis of specific n-7 versus n-9 MUFA species [24].

This study is limited by the availability of commercial cells i.e. neonatal and adult NHEK and potential for gender bias. Therefore, we undertook principal component analysis which confirmed that age rather than gender was the major influence on FA profile and methylation.

In conclusion, novel PUFA profile differences between adult and neonatal NHEK are associated with lower activity in the n-7, n-9 and n-10 FA biosynthetic pathways and desaturation via FADS2. Lower FADS2 gene expression was associated with hypermethylation of the FADS2 promotor at the binding site of the helicase SMARCA4. Further work is needed to understand the contribution of epigenetic regulation at the SMARCA4 binding site for barrier function in health and disease.

## Author contributions

HRG and GJ conceived the project. CP, DH, DM acquired the data, assisted by LJW, KEB, DT, MIF and FLL for analysis; CP and HRG wrote the core manuscript text and all authors contributed to revising it critically for important intellectual content and final approval of the version to be submitted.

## Competing financial interests statement

None.

## Declaration of competing interest

Messenger D.J.^2^, Barrett K.E.^2^, Hyliands D.^2^, Talbot D.^2^, Fowler M.I.^2^, Kawatra T.^2^, Gunn D.A.^2^, Lim FL.^2^, Wainwright L.J.^2^, Jenkins G.^2^, are employed by Unilever R&D.

Griffiths H.R.^1,3^ received part funding from 10.13039/100007190Unilever R&D and 10.13039/501100000268BBSRC to undertake this study.
