# Supplementary material for: Lower polyunsaturated fatty acid levels and FADS2 expression in adult compared to neonatal keratinocytes are associated with FADS2 promotor hypermethylation
Source: Biochem Biophys Res Commun. 2022 Apr 23;601:9–15. doi: 10.1016/j.bbrc.2022.02.055 (PMC8993048; doi:10.1016/j.bbrc.2022.02.055)
Supplement: Multimedia component 1 [file mmc1.docx]

**Supplementary Information**

A
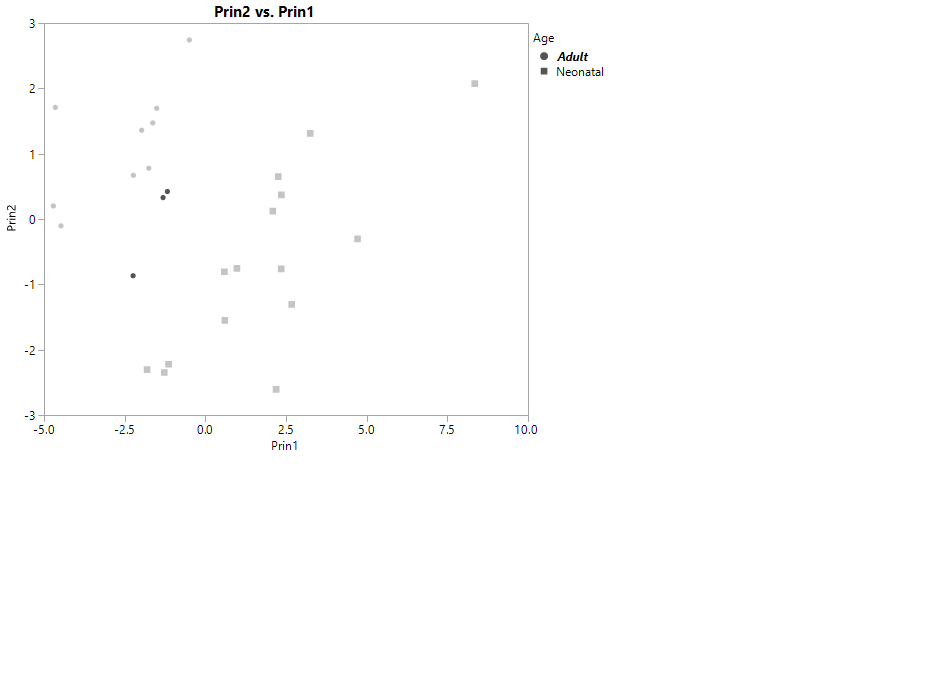


B
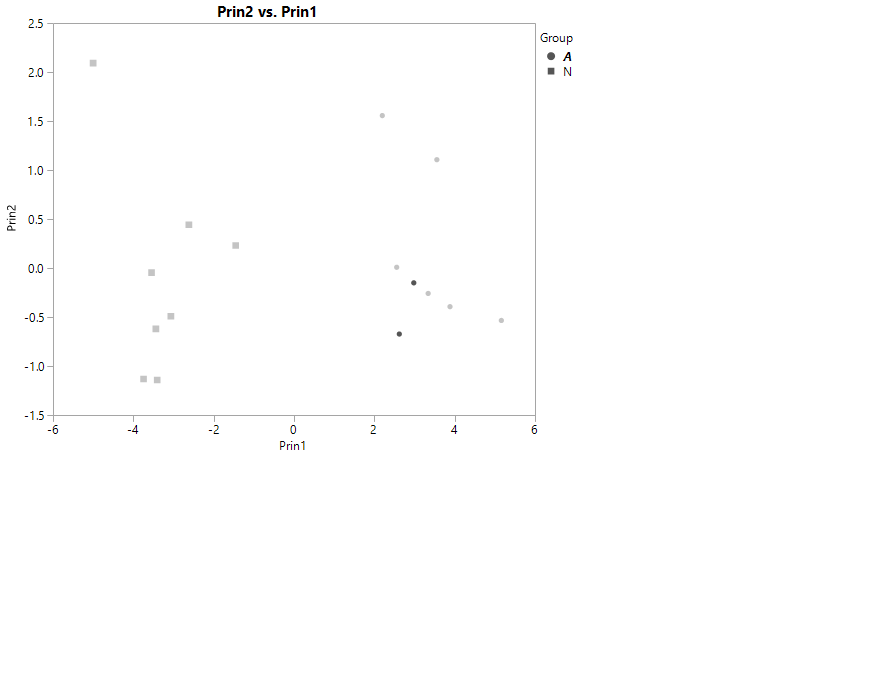


Supplementary Figure 1: A. Principal (Prin) component analysis of neonatal and adult keratinocyte FA profiles in triplicate.  B. Principal component analysis of neonatal (N) and adult (A) keratinocyte FADS2 methylation profiles in duplicate.  Both datasets were analysed by t-test and filtered to keep only entities which were significantly different (p<0.05) between neonatal and adult samples.  Highlighted points (black) in both datasets show the adult male samples.


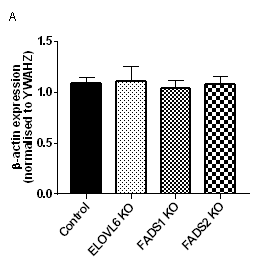
 
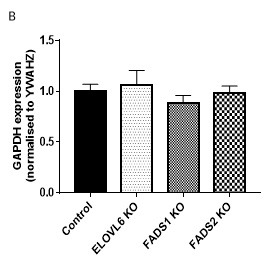


Supplementary Figure 2. A. β-actin AND B. GAPDH expression was unaffected in neonatal keratinocytes after targeting siRNA to reduce ELOVL6, FADS1 or FADS2 enzyme expression.
