# Supplementary material for: Lower polyunsaturated fatty acid levels and FADS2 expression in adult compared to neonatal keratinocytes are associated with FADS2 promotor hypermethylation
Source: Biochem Biophys Res Commun. 2022 Apr 23;601:9–15. doi: 10.1016/j.bbrc.2022.02.055 (PMC8993048; doi:10.1016/j.bbrc.2022.02.055)
Supplement: Multimedia component 2 [file mmc2.docx]

Supplementary Table 1

|  | Housekeeper (YWAHZ) | | | Keratin-7 | | |
| --- | --- | --- | --- | --- | --- | --- |
| A | 18.85 | 18.58 | 18.67 | 30.58 | 30.52 | 31.02 |
| B | 18.78 | 18.33 | 18.67 | 30.28 | 30.31 | 30.19 |
| C | 18.36 | 18.38 | 18.19 | 30.45 | 30.38 | 30.16 |
| D | 18.48 | 18.41 | 18.53 | 32.35 | 32.57 | 32.19 |
| E | 18.58 | 18.47 | 18.23 | 31.11 | 31.27 | 31.33 |
| F | 18.67 | 18.68 | 18.57 | 31.75 | 31.64 | 31.48 |
| G | 18.37 | 18.56 | 18.19 | 31.48 | 30.27 | 32.19 |
| H | 18.28 | 18.34 | 18.72 | 34.09 | 34.91 | 35.61 |
| I | 18.82 | 18.76 | 18.68 | 36.01 | 34.21 | 31.68 |
| J | 18.35 | 18.38 | 18.77 | 34.29 | 32.68 | 32.58 |
| K | 18.27 | 18.36 | 18.44 | 34.11 | 33.56 | 33.98 |

Ct values describing expression of sebocyte marker keratin 7 in proliferating adult and neonatal NHEK
